# The Surgical Management of Lung Neuroendocrine Neoplasms

**DOI:** 10.3390/cancers15061695

**Published:** 2023-03-09

**Authors:** Patrick Soldath, René Horsleben Petersen

**Affiliations:** 1 Department of Cardiothoracic Surgery, Rigshospitalet, 2100 Copenhagen Ø, Denmark; 2 Department of Clinical Medicine, University of Copenhagen, 2200 Copenhagen N, Denmark

**Keywords:** lung neuroendocrine neoplasms, carcinoid, large cell neuroendocrine carcinoma, small cell lung cancer, surgery

## Abstract

**Simple Summary:**

Lung neuroendocrine neoplasms comprise a diverse group of cancers that arise from pulmonary neuroendocrine cells. They account for about 20% of all primary lung cancers and classify into four subtypes: typical carcinoid, atypical carcinoid, large cell neuroendocrine carcinoma, and small cell lung carcinoma. These subtypes share some morphological and protein expression immunohistochemistry features but differ greatly in their biological behaviors. Typical and atypical carcinoids are well differentiated low- and intermediate-grade tumors, respectively, whereas large cell neuroendocrine carcinoma and small cell lung carcinoma are poorly differentiated high-grade tumors. The common thread in all lung neuroendocrine neoplasms is that patients with localized disease primarily undergo surgery, while patients with locally advanced or metastatic disease receive tailored oncological therapies.

**Abstract:**

This review summarizes key recent developments relevant to the surgical management of lung neuroendocrine neoplasms (L-NENs), including typical and atypical carcinoids, large cell neuroendocrine carcinoma, and small cell lung carcinoma. This review includes recent insights into the classification, clinical presentation, diagnostic workup, treatment options, and follow-up. Highlighted topics include general principles of surgery in localized or locally advanced or metastatic L-NENs, lung-sparing surgery for small, peripheral typical carcinoids, adjuvant and systemic therapies for typical and atypical carcinoids, and surgery and adjuvant therapies for large cell neuroendocrine carcinoma and small cell lung carcinoma.

## 1. Introduction

Neuroendocrine neoplasms (NENs) comprise a diverse group of cancers that arise from specialized, peptide- and amine producing cells dispersed throughout the diffuse endocrine system. They most frequently occur in the gastrointestinal tract (48%), lung (25%), and pancreas (9%) but may also develop in many other organs, including the breast, prostate, thymus, and skin [1]. Lung NENs (L-NENs) account for about 20% of all primary lung cancers and encompass neuroendocrine tumors (L-NETs) composed of typical carcinoid (TC, 1.8%) and atypical carcinoid (AC, 0.2%) and neuroendocrine carcinomas (L-NECs) composed of large cell neuroendocrine carcinoma (LCNEC, 3%) and small cell lung carcinoma (SCLC, 15%). L-NETs and L-NECs are thought to represent distinct and separate entities with neither molecular overlap nor a common developmental continuum [2].

The mainstay of treatment for localized L-NENs is radical resection and systematic nodal dissection. The recommended resections have traditionally been lobectomy, bilobectomy, or pneumonectomy, depending on the location and size of the tumor. However, in recent years, a debate has emerged about whether lung-sparing resections such as sublobar and sleeve resections could replace the traditional resections in selected L-NET cases. On the other hand, the optimal treatment for advanced or metastatic disease is still to be determined. The current options include somatostatin analogs (SSAs), targeted therapy, and peptide receptor radio-targeted therapy (PRRT) for L-NETs, as well as various chemotherapy regimens for both L-NETs and L-NECs. Nevertheless, surgery also takes its place in the setting of locally advanced or metastatic disease, as, for example, bronchoscopic procedures can prevent obstruction of airways from central tumors and surgical debulking can improve control of secretory symptoms from large tumors.

This review summarizes the classification, presentation, diagnostic workup, treatment, and follow-up of L-NENs, focusing on the role of surgery in both localized and locally advanced or metastatic disease.

## 2. Classification

In the current *World Health Organization (WHO) 2021 classification (fifth edition)*, L-NENs comprise four subtypes, which share some morphologic and protein expression immunohistochemistry (IHC) features but differ greatly in their biological behaviors: TC and AC are well-differentiated low-grade and intermediate-grade tumors, respectively, whereas LCNEC and SCLC are poorly differentiated high-grade tumors [3]. The WHO classification makes a clear distinction between well-differentiated and poorly-differentiated tumors by referring to TC and AC as L-NETs and LCNEC and SCLC as L-NECs. This distinction is based on recent molecular studies indicating that L-NETs and L-NECs have disparate genomic profiles and therefore must be considered as distinct and separate entities [4].

**Figure 1:**
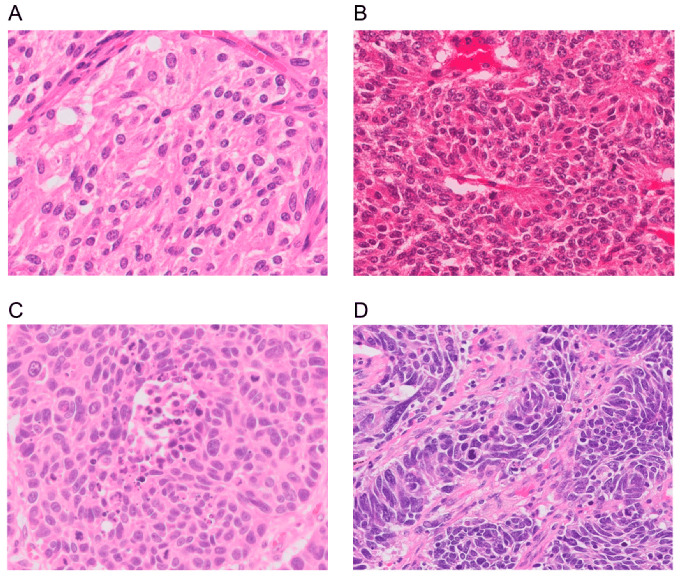
Morphology of a lung neuroendocrine neoplasm. (**A**) A typical carcinoid showing solid nests with zellballen patterns; the tumor cells are uniform with a moderate amount of eosinophilic cytoplasm. (**B**) An atypical carcinoid showing rosette formation. (**C**) Large-cell neuroendocrine carcinoma showing organoid nesting and palisading patterns; tumor cells have abundant eosinophilic cytoplasm, coarsely granular chromatin, and prominent nucleoli. (**D**) Small-cell lung carcinoma showing sheets of small cells with scant cytoplasm, finely granular chromatin, and mitoses. Magnification: ×40. Adapted from Yoshimura et al. [5].

The diagnostic criteria of L-NENs are based on characteristic neuroendocrine morphology and IHC markers (Chromogranin A [CgA], synaptophysin, and CD56/neural cell adhesion molecule [NCAM]) in combination with the mitotic rate per 2 mm^2^ and the absence or presence of necrosis. TC has <2 mitoses/2 mm^2^ and no necrosis, AC has 2–10 mitoses/2 mm^2^ and focal necrosis, and LCNEC and SCLC both have >10 mitosis/2 mm^2^ and abundant necrosis but differ in cell size and nuclear features. However, it is important to recognize that because L-NETs and L-NECs are different entities, the criteria of mitotic rate and degree of necrosis cannot be used to distinguish between them. Instead, the key distinguishing feature between L-NETs and L-NECs is their distinct overall morphology. Figure 1 shows the morphology of the four subtypes [5].

L-NENs follow the tumor-node-metastasis (TNM) system and stage groupings for lung cancers described in the recent eighth edition of *The American Joint Committee on Cancer (AJCC) Staging Manual*, which focuses on the size and invasion of the tumor [6]. However, for practical purposes, L-NENs can be considered either localized disease (radically resectable) or locally advanced or metastatic disease (unresectable).

## 3. Clinical Presentation

L-NENs can present in many ways, primarily depending on the subtype, location, and size of the tumor. TC occurs in adults around 45 years of age on average (and may even occur in children and adolescents), which is earlier than AC (~55 years) and LCNEC/SCLC (~65 years) [7,8]. The majority of TC patients are nonsmokers, whereas AC appears to be slightly more frequent in smokers. Conversely, LCNEC and SCLC are almost exclusively related to cigarette smoking, with more than 90% of the patients being heavy smokers [9].

Typically, L-NETs grow slowly and metastasize late, if at all, and thus can be asymptomatic for a long time. In contrast, L-NECs grow aggressively and metastasize early, which can cause both respiratory and general symptoms to present shortly after the tumor has arisen. Central tumors tend to present with obstructive symptoms related to the tumor mass, such as cough, wheezing, hemoptysis, dyspnea, chest pain, and recurrent pulmonary infections [10,11,12]. All these symptoms often mimic other diseases like COPD or asthma, which may delay diagnosis. Peripheral tumors are often asymptomatic and found incidentally, but they may also present with one or more of the symptoms that are seen in central tumors.

Albeit rare, L-NETs can cause a variety of symptoms and syndromes from secreted hormones that reach systemic circulation. Some of the most common presentations include Cushing’s syndrome, acromegaly, and hypoglycemia caused by the secretion of adrenocorticotrophic hormone (ACTH), growth hormone-releasing hormone (GRHR), and insulin-growth factors (IGF), respectively [12,13,14,15,16,17,18,19,20]. Therefore, patients with unexplained Cushing’s syndrome, acromegaly, or hypoglycemia should be evaluated for L-NETs. Another presentation is carcinoid syndrome, which is caused by the secretion of various hormones (primarily serotonin) and is characterized by flushing, diarrhea, and carcinoid heart disease. It occurs in about 2–5% of patients with L-NETs and mainly in patients with liver metastasis, allowing for bypass of hepatic metabolism that may inactivate the hormones [7]. Carcinoid heart disease (CHD) is a feared complication in patients with long-standing carcinoid syndrome [21,22,23]. It occurs in about half of patients with carcinoid syndrome and is characterized by pathognomonic fibrotic plaques on heart valves, leaflets, and papillary muscles. Usually, it affects the tricuspid and pulmonic valves the most and can lead to valvular dysfunction and, in severe cases, right-sided heart failure [24]. Finally, carcinoid syndrome can turn into an acute, life-threatening condition known as carcinoid crisis. This condition is characterized by profound flushing, bronchospasm, and rapidly fluctuating blood pressure. It may be precipitated by the induction of anesthesia or palpation, ablation, or embolization of an L-NET [25]. Therefore, patients with carcinoid syndrome should be given a somatostatin analogue before any anesthetic or tumor manipulation [7].

Like L-NETs, SCLC can cause Cushing’s syndrome in about 5% of patients [26]. Two other common syndromes in SCLC are syndrome of inappropriate antidiuretic hormone secretion (SIADH) and Lambert-Eaton myasthenic syndrome, which occur in about 10% and 3% of patients, respectively [27,28]. In contrast to L-NETs and SCLC, LCNEC is not associated with any paraneoplastic endocrine syndromes except for extremely rare case reports of Cushing’s syndrome and SIADH [29,30].

## 4. Diagnostic Workup

Patients suspected of having L-NENs should be discussed within a multidisciplinary tumor board involving specialists from pulmonology, thoracic surgery, medical oncology, radiology, nuclear medicine, and pathology. The diagnosis should be based on results from biochemical, imaging, and pathological studies.

### 4.1. Biochemical Studies

Patients with symptoms of hormonal hypersecretion (e.g., Cushing’s syndrome or carcinoid syndrome) should undergo a complete evaluation and workup with input from endocrinology. Biochemical testing should be targeted to the presenting syndrome, which in the case of Cushing’s syndrome and carcinoid syndrome includes serum levels of cortisol, ACTH, and serotonin as well as a 24 h urine 5-hydroxyindole acetic acid (5-HIAA) test.

Patients without symptoms of hormonal hypersecretion should not routinely undergo biochemical testing. However, it may be useful to obtain a baseline CgA, which can be tracked during treatment if it is abnormal [31].

### 4.2. Anatomical and Functional Imaging

**Figure 2:**
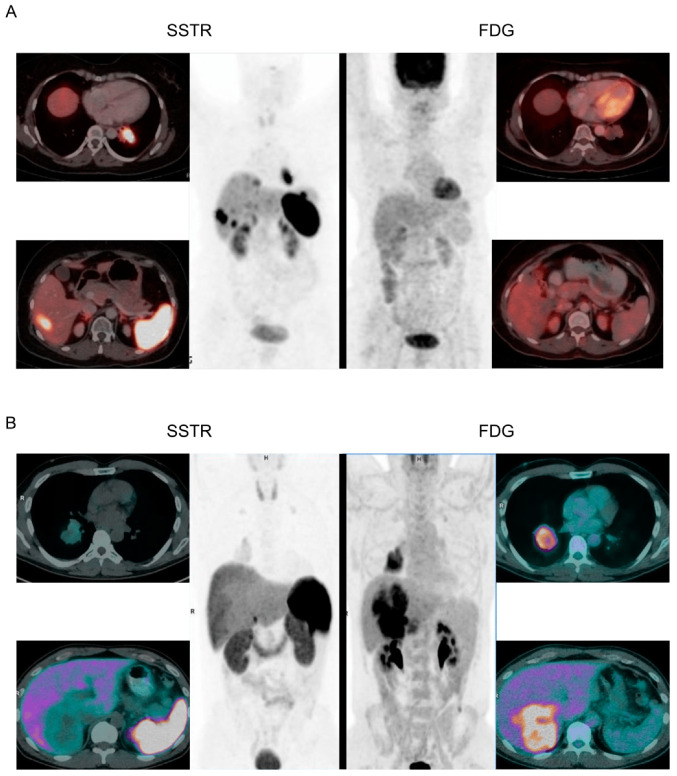
Functional scans of L-NETs and L-NECs. (**A**): A patient with L-NET was scanned with both a somatostatin analog (SSTR) scan and a glucose analog scan (FDG). The SSTR scan shows high uptake of somatostatin in the primary tumor and in a liver metastasis, while the FDG scan shows no uptake of FDG in the primary tumor and does not reveal the liver metastasis. (**B**): A patient with L-NEC was scanned with both an SSTR scan and an FDG scan. The FDG scan shows high uptake of FDG in the primary tumor and in a large conglomerate of liver metastases, while the SSTR scan shows no uptake of somatostatin in the primary tumor and does not reveal the liver metastases. Adapted from Purandara et al. and Chan et al. [32,33].

Patients should undergo both an anatomical and a functional scan for clinical staging. The anatomical scan should be a contrast-enhanced computed tomography (CT) of the chest and either a multiphase CT or magnetic resonance imaging (MRI) of the liver, while the functional scans can be either a somatostatin analog scan (e.g., ^64^Cu-/^68^Ga-DOTATATE/-TOC/-NOC positron emission tomography [PET], ^111^In-octreotide scintigraphy, or ^123^I-MIBG scintigraphy) or a glucose analog scan (e.g., ^18^F-FDG PET). Somatostatin analog scans take advantage of the overexpression of somatostatin receptors, which is commonly seen in L-NETs but not in NECs. In contrast, glucose analog scans take advantage of the increased glucose metabolism of cancer cells, which is more pronounced in L-NECs than in L-NETs. Therefore, somatostatin analog scans are recommended for patients suspected of L-NETs, while glucose analog scans are recommended for patients suspected of L-NECs. Figure 2 illustrates the importance of performing the correct functional scan in L-NETs and L-NECs [32,33].

On both anatomical and functional scans, L-NETs typically present as a smooth, rounded, homogenous nodule or mass within the lung parenchyma or in an endobronchial location with associated postobstructive atelectasis or air trapping [34].

L-NETs are well perfused and thus have a high uptake of contrast medium, helping to differentiate them from benign nodules, which usually show only a low contrast medium uptake. In contrast, L-NECs typically present as large peripheral (LCNEC) or central masses (SCLC) with extensive lymphadenopathy, pleural carcinosis, and direct mediastinal invasion [35,36,37]. In both L-NETs and L-NECs, CT or MRI in combination with either a somatostatin analog scan (L-NETs) or a glucose analog scan (L-NECs) are excellent to detect lymphadenopathy and metastatic disease to the most common sites, including bone, brain, liver, or adrenal glands [36].

### 4.3. Luminal Imaging

Flexible bronchoscopy is a relatively non-invasive procedure that allows for direct visual examination and tissue sampling of central tumors. On bronchoscopy, central L-NETs appear as strongly vascularized masses covered by bronchial epithelium. However, often they extend well beyond the inside of the airways (the so-called iceberg phenomenon) [38]. Thus, the bronchoscopic findings must be evaluated in relation to the anatomical and functional imaging studies. In contrast, central L-NECs often appear more irregular and with surrounding infiltration. For more peripheral tumors, electromagnetic navigational bronchoscopy may be used for tissue sampling [39]. In rare cases (<1%), tissue sampling can cause significant bleeding from the tumor, which can be managed with endobronchial interventions such as cryotherapy, epinephrine injection, or Nd:YAG laser [40]. Endobronchial ultrasound-guided transbronchial needle aspiration (EBUS-TBNA) or mediastinoscopy is recommended for staging according to the TNM classification for lung cancer. Although EBUS-TBNA is mainly useful for detecting nodal involvement and cannot reliably differentiate between TC and AC.

### 4.4. Echocardiography

Echocardiography can be used to identify CHD. In CHD, 90% of cases show right atrial and ventricular enlargement, and 50% show ventricular septal wall abnormalities [24]. The tricuspid valve leaflets and subvalvular structures are often thickened, shortened, and retracted, which leads to incomplete valve closure and, usually, moderate or severe tricuspid regurgitation. However, about half of patients with CHD are asymptomatic, stressing the importance of screening echocardiography in patients with L-NETs to allow timely intervention in valvular disease [22]. If signs of CHD are found on echocardiography, further imaging with velocity-encoded cine MRI can be performed to precisely assess and quantify the motion and dysfunction of the valves [41].

### 4.5. Pathology

Obtaining tissue for pathology testing is mandatory for the diagnosis of L-NENs. When surgical resection is not an option, core needle biopsy (transbronchial or transthoracic) is preferred over fine needle aspiration to allow full assessment of the tumor architecture.

## 5. Treatment Options

Patients diagnosed with L-NENs should be rediscussed within the multidisciplinary tumor board and receive an individualized treatment plan based on tumor factors such as subtype, stage, and clinical presentation, as well as demographic factors such as age and comorbidities. As far as possible, treatment plans should conform to applicable guidelines. Currently, five sets of guidelines exist for L-NETs (ENETS, NANETS, CommNETS-NANETS, ESMO, and NCCN), and three sets exist for SCLC (ESMO, NCCN, and ASCO) [7,42,43,44,45,46,47]. Because L-NETs are rare and heterogenous, guidelines are based on merely low-quality data and medium-strength evidence. No guidelines exist for LCNEC, so it is commonly treated based on the genomic profile of the tumor. Tumors that express genetic patterns most compatible with SCLC (SCLC-like LCNEC) are treated according to the SCLC guidelines, and tumors that are more like non-small cell lung cancer (NSCLC; NSCLC-like LCNEC) are treated according to the guidelines for NSCLC (ESMO and NCCN) [48,49,50]. All guidelines recommend surgical resection as the primary treatment in patients with localized disease and systemic treatment as the primary treatment in patients with locally advanced or metastatic disease. Commonly, L-NETs present with localized disease due to their low malignant potential, whereas L-NECs present with locally advanced (extensive lymphadenopathy and direct mediastinal invasion) and/or metastatic disease due to their high malignant potential.

### 5.1. General Principles for Surgical Management of L-NENs

Surgically fit patients with localized L-NENs should be managed with radical resection and systematic nodal dissection. The most important objective is a microscopically tumor-free resection margin (R0), which is associated with a good prognosis and the best outcomes in all subtypes [51,52,53,54,55,56,57,58,59,60,61,62,63]. The 10 year survival rate for TC is more than 90% in lymph node-negative disease (N0) and is still up to 75% if either hilar (N1) or mediastinal (N2) lymph nodes are affected. Therefore, surgery is recommended in TC for stages N0–N2. The 10 year survival rate for AC is 60% in N0 disease but drops to 50% and 30% in N1 and N2 stages, respectively. Nevertheless, surgery is also recommended in AC for stages N0–N2. On the other hand, the 5 year survival rate for LCNEC and SCLC is 30–60% in N0 disease but a mere 5–10% in stage N1 or N2. Therefore, surgery is generally only recommended in LCNEC and SCLC for stages N0–N1.

**Figure 3:**
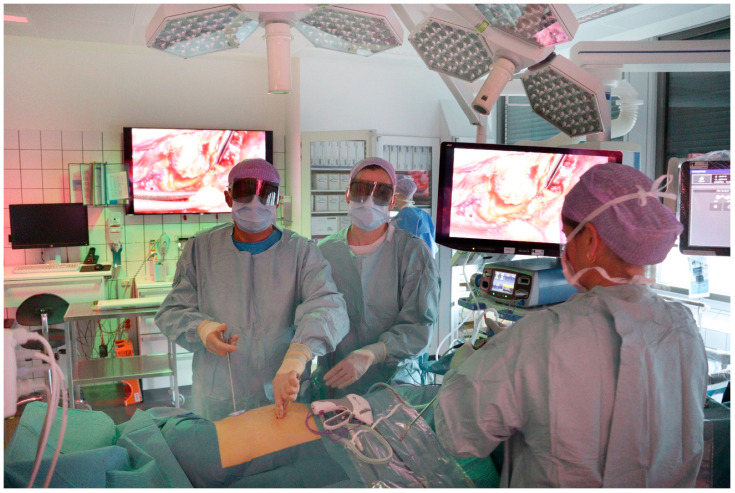
Modern VATS setup with advanced endoscopic instruments, 4K 3D monitors, and polarized 3D glasses.

The standard resection is an anatomical resection (lobectomy, bilobectomy, or pneumonectomy), depending on the location and size of the tumor. However, a sublobar resection (wedge or segmentectomy) may be performed in the small peripheral TC [43]. In central tumors, lung-sparing resections such as bronchial sleeve resection and sleeve lobectomy are preferred over bilobectomy and pneumonectomy due to lower morbidity and better survival rates [64,65,66]. The systematic nodal dissection should conform to the guidelines of the International Association for the Study of Lung Cancer (IASLC), which require the resection of a minimum of six nodes/stations, three of which should be mediastinal, including the subcarinal station [67]. A minimally invasive approach, such as video-assisted thoracoscopic surgery (VATS) or robot-assisted thoracic surgery (RATS), is recommended in experienced centers due to its benefits of less pain, better physical function, a shorter length of stay, fewer complications, and potentially increased survival rates [68,69,70,71,72]. Even technically complex procedures such as sleeve lobectomy can be managed with VATS or RATS with comparable feasibility and short-term outcomes of morbidity and mortality to an open approach [73,74]. Figure 3 shows a modern VATS setup with advanced endoscopic instruments, 4K 3D monitors, and polarized 3D glasses.

### 5.2. Localized L-NETs

All guidelines recommend surgical resection as the primary treatment of choice for localized L-NETs and consider it the only curative option.

#### 5.2.1. Surgery

The recommended surgical management for L-NETs depends on the subtype, location, and size of the tumor. Peripheral L-NETs (i.e., without involvement of the central bronchi) are recommended as an R0 resection through a lobectomy. However, the latest CommNETs-NANETS guidelines proposed that peripheral TC without nodal involvement and less than 2 cm in size (stage I) can be managed with a sublobar R0 resection [43]. This new recommendation is based on recent large registry-based studies, which found no difference in the 5 year survival rate between lobectomy (88%) and sublobar resection (87%) for small peripheral TC [75,76,77]. Three subsequent studies have gone a step further and investigated whether there is a difference in survival between segmentectomy and wedge resection. Two of those studies found no difference in survival between lobectomy (94%), segmentectomy (94%), and wedge resection (90%), but the last study found a significantly worse survival for wedge resection (94%, 96%, and 82%; *p* < 0.001) [78,79,80]. A fourth study included peripheral TC up to stage III and found no differences in survival between lobectomy and sublobectomy; however, it did not compare survival between segmentectomy and wedge resection [81]. Moreover, two recent large, noninferiority trials found comparable survival between lobectomy and sublobectomy in peripheral NSCLC (squamous cell carcinoma, adenocarcinoma, and others) without lymph node involvement and less than 2 cm in size (stage IA) [82,83]. The first trial randomized 1106 patients 1:1 to either lobectomy or segmentectomy and found 5 year disease-free survival rates of 88.0 and 87.9%, respectively, while the second trial randomized 697 patients 1:1 to either lobectomy or sublobectomy and found 5 year disease-free survival rates of 64.1 and 63.6%, respectively. Both studies concluded that sublobar resection was noninferior to lobectomy for disease-free survival. Furthermore, both studies found that the patients who underwent sublobar resection had significantly less decline in lung function than those who underwent lobectomy, although not as pronounced as expected. Based on the studies of small peripheral L-NETs and NSCLC, a sublobar resection seems to be a reasonable treatment for peripheral stage I TC.

An important aspect of sublobar resections is that they prevent an extensive dissection of N1 lymph nodes and thus carry a risk of missing positive N1 lymph nodes between the resection site and the dissected N2 lymph nodes. In fact, one of the supporting studies for the new recommendation found a significantly higher proportion of unforeseen positive N1 lymph nodes between lobectomy (5.2%) and sublobectomy (0.7%) [75]. The topic of lobectomy and sublobectomy in peripheral stage I TC remains a matter of debate, and for now, it is only endorsed by the CommNETs-NANETS guidelines.

Peripheral AC is always recommended for a lobar R0 resection, independent of tumor size, due to its higher malignant potential.

For central NETs, the surgical objective is parenchymal-sparing surgery. Where possible, bronchial sleeve resection (no lung tissue is removed) or a sleeve lobectomy should be carried out in preference to a pneumonectomy (with intraoperative frozen section analysis of the resection margins). This recommendation is based on a few minor but forceful studies that have found comparable survival and recurrence between parenchymal-sparing surgery and traditional lobar resections [84,85,86,87,88,89,90,91]. Because central AC is particularly rare, it was poorly represented in the studies, and thus the evidence for parenchyma-sparing surgery in AC is weaker. A large registry-based study on TC reported comparable 10 year disease-free survival rates for 2876 patients who underwent lobectomy (97%) and 929 who underwent sublobectomy (98%) [92].

Completely intraluminal NETs (without extension through the cartilaginous wall) can be bronchoscopically resected (using Nd:YAG laser, diathermy, or cryotherapy) with comparable survival to bronchial sleeve resection [38,93,94,95,96,97,98,99]. In the event of residual disease or unforeseen extraluminal involvement, surgical resection can be performed following usual guidelines [38]. Moreover, bronchoscopic resection is a good alternative for patients who are unfit for surgery, but it comes with important drawbacks such as no lymph node staging and a need for careful follow-up with repeated bronchoscopy to examine for possible recurrence [38,94,98].

If local recurrence occurs after either surgical or bronchoscopic resection, it is recommended to redo the surgical resection following the same guidelines as for the primary surgery.

Surgically unfit patients (i.e., those with significant comorbidities or a high operative risk) can be considered for stereotactic body radiation therapy (SBRT). In cases where both surgery and SBRT are contraindicated, radiofrequency ablation (RFA) can be considered. Finally, watchful follow-up with serial diagnostic imaging can be considered in patients with low-volume, asymptomatic, and nonfunctional NETs, especially TC, as a large study of 306 patients showed that many of those patients remain well without disease progression for years [92].

#### 5.2.2. Adjuvant Treatment

The NCCN guidelines recommend adjuvant therapy (platinum-based etoposide with or without radiation) based on subtype, stage, and state of resection margins. AC is recommended as adjuvant therapy from stage IIIA regardless of resection margins, while TC is recommended as adjuvant therapy from stage IIIA if resection margins are positive and surveillance if resection margins are negative. The ESMO guidelines recommend an individualized adjuvant treatment plan for AC with positive lymph nodes on a patient-by-patient basis after a multidisciplinary evaluation, while the NANETS and CommNETs-NANETS guidelines recommend against adjuvant systemic treatment due to a lack of clinical evidence.

Adjuvant radiation is recommended in AC in stage N2, but the survival benefit has not been established in a high-quality randomized study.

### 5.3. Locally Advanced or Metastatic L-NETs

In locally advanced and metastatic L-NETs, a multidisciplinary approach should be taken with the goal of providing palliative care. These cases are usually considered incurable, and treatment aims to control secretory symptoms and inhibit tumor growth.

#### 5.3.1. Locoregional Therapy

For locally advanced L-NETs, definitive radiation therapy (RT) in combination with chemotherapy (typically a platinum-based doublet) is recommended. This modality is extrapolated from regimens used for NSCLC and SCLC, and the optimal choice and composition of RT and chemotherapy are not yet settled.

For metastatic L-NETs, palliative RT is recommended for symptomatic tumors, while RFA is recommended for liver, lung, or bone metastases.

#### 5.3.2. Systemic Therapy

All guidelines recommend systemic therapies, including SSAs (e.g., octreotide or lanreotide), targeted therapy (e.g., everolimus), PRRT (e.g., ^90^Yttrium or ^117^Lutetium), and chemotherapy (e.g., platinum-based doublet or temozolomide).

SSAs play two key roles in L-NETs: they control the symptoms of carcinoid syndrome and inhibit the proliferation of tumor cells. A recent RCT found a survival benefit in patients treated with lanreotide (16.6 months) against patients treated with placebo (13.6 months) [100]. Two other small studies also found a clinically meaningful survival benefit in patients treated with SSAs [101,102]. Further evidence for the use of SSAs in L-NETs relies on studies of gastroenteropancreatic NETs (GEP-NETs) that found significant survival benefits from SSAs [103,104].

Targeted therapy also plays a role in L-NETs, although only everolimus has been firmly shown to improve survival in L-NETs. A large RCT randomized 90 patients with L-NETs (a subgroup of 302 total patients with NETs of different sites) to either everolimus or placebo and found a significant benefit on progression-free survival in patients treated with everolimus (11.0 months) against patients treated with a placebo (3.9 months) [105]. Another RCT randomized 124 patients with L-NETs into three treatment groups: pasireotide (an SSA), everolimus, or the combination, and found a significantly higher proportion of patients who were progression-free at 9 months after treatment in the combination group (39%, 33%, and 59%, respectively) [106]. Together, these studies show that everolimus is a valuable treatment option for patients with L-NETs.

Chemotherapy is recommended for metastatic L-NETs, but this recommendation is based on small and mainly retrospective trials. Several trials have investigated the effect of platinum-based doublet chemotherapy in mixed populations of NETs that only included a minor number of L-NETs. These trials found varying responses ranging from 0 to 33%, suggesting that platinum-based doublet chemotherapy is not as effective in L-NETs as in L-NECs [107,108,109,110,111]. Other studies examined temozolomide either alone or in combination with other agents such as bevacizumab or capecitabine and found a partial response in 12–33% of patients and stable disease in 52–74%, with the best results for the double therapy regimens [112,113,114,115,116,117]. Another study investigated oxaliplatin combined with either 5-fluorouracil or gemcitabine in L-NETs and found comparable results, with a partial response in 20% of patients and stable disease in 64%, with no significant differences between either regimen [118]. All these studies indicate that several different chemotherapy options may be suitable in L-NETs with reasonably satisfactory results.

L-NETs that express somatostatin receptors (~80%) may benefit from PRRT. PRRT utilizes the ability of L-NETs to bind radioactive β -emitter-labelled SSA to somatostatin receptors (SSTR2) on tumor cells and thereby deliver cytotoxic radiation. This approach is primarily indicated as second- or third-line treatment in locally advanced or metastatic L-NETs with a tumor uptake higher than the physiological liver uptake at somatostatin imaging [119]. The goal of therapy is palliative and aimed at prolonging progression-free survival and reducing symptoms, with a 5% chance of a complete response and up to a 50% chance of a partial response [120]. The radiopharmaceuticals used for PRRT are ^177^Lu-DOTATATE and ^90^Y-DOTATOC. The first option may be more effective for smaller tumors, while the latter may be more effective for larger tumors due to their different beta-decay ranges [121]. Studies have shown that a partial response is achieved in 20–25% of the patients, and more than 60% achieve stable disease [122,123,124,125]. However, prospective trials are needed to determine the optimal number of cycles, doses, and timing for PRRT.

#### 5.3.3. Surgery

Locally advanced L-NETs that invade and obstruct the central airways are recommended for bronchoscopic resection to disobliterate the airways using an appropriate method such as Nd:YAG laser, cryotherapy, or argon beaming. Moreover, in selected cases, a bronchial stent may be placed to keep the airways from becoming obstructed again or to cover a fistula.

Metastatic L-NETs with large tumor volumes and secretory symptoms that are not effectively managed by SSAs are recommended for surgical debulking if technically feasible to improve control of secretory symptoms. Retrospective studies suggest that aggressive treatment of liver metastases in L-NET patients can lead to better survival rates, with 5 year survival rates as high as 61–94% in selected patients [126,127,128,129,130,131]. Nevertheless, surgery and local ablative therapies such as SBRT, RFA, and microwave ablation are only possible in less than 10% of patients due to the extent of the disease [126,130]. When multiple liver metastases are present, selective palliative treatments like transcatheter arterial bland embolization or chemoembolization and selective internal RT treatment of liver metastases may be considered [132]. Even if radical resection is not possible, patients with metastatic disease in the liver (up to 75% of liver volume) should be evaluated for debulking, especially if hormone-related symptoms are difficult to control [119].

### 5.4. Localized L-NECs

Localized LCNEC follows the guidelines of NSCLC with surgical resection for stages I–IIIA, while SCLC follows its own guidelines with surgical resection solely for stage I. Surgical resection in both LCNEC and SCLC should always be combined with neoadjuvant and/or adjuvant therapy.

#### Surgery for L-NECs

Because LCNEC is a rare tumor and difficult to distinguish from SCLC on small (preoperative) biopsies, LCNEC has been particularly challenging to investigate in large, prospective trials. However, small, retrospective studies suggest that LCNEC should be managed with a lobar R0 resection for stages I–IIIA in combination with neoadjuvant and/or adjuvant therapy, which results in 5 year survival rates ranging between 27 and 67% [58,133,134,135]. A recent large, registry-based study investigated 6092 patients with LCNEC in stages I, II, and III and found overall 5 year survival rates of 50, 45, and 36%, respectively, with significant survival benefits for patients treated with adjuvant therapy across all stages [136]. Similar findings were reported by another large registry-based study [137]. The first mentioned registry-based study also found significantly better 5 year survival rates in stage I patients undergoing surgery and adjuvant therapy (50%) compared with SBRT (27%), and stage II and IIIA patients undergoing surgery and adjuvant therapy (between 45 and 36%) compared with chemoradiation (between 12 and 25%). This superior survival of surgery compared with SBRT in stage I LCNEC has been confirmed by two subsequent registry-based studies [138,139]. Another recent large, registry-based study investigated 2642 patients with stage I LCNEC and found a significant survival benefit in patients treated with lobectomy compared with sublobar resection for both stage IA (HR: 0.72, *p* < 0.001) and stage IB (HR: 0.63, *p* = 0.020) [140]. This superior survival of lobectomy compared with sublobar resection has been confirmed by other studies [141,142,143,144]. The study also found a significant survival benefit in patients treated with adjuvant therapy for stage IB (HR: 0.67, *p* = 0.007) but not for stage IA (HR: 0.92, *p* = 0.429). Several studies have evaluated chemotherapy in LCNEC, but the reported studies are heterogeneous in case selection and confirmation of the pathology diagnosis. However, a recent study, which presented the largest series of patients with pathology-reviewed metastatic LCNEC to date, found that NSCLC-like regimens, mainly platinum-gemcitabine, are superior to the common SCLC-like regimen of platinum-etoposide (HR: 1.66, *p* = 0.020) [145].

In conclusion, localized LCNEC in stages I–IIIA should be managed with a surgical R0 lobar resection combined with neoadjuvant and/or adjuvant therapy (preferably NSCLC-like, e.g., platinum-gemcitabine).

The old tenacious fact that surgery plays no role in SCLC was determined in 1975 and ratified in 1994, when the only two randomized controlled trials that have been conducted in SCLC reported that surgical resection had no effect on survival and was even inferior to RT [146,147]. However, both of these studies suffered from important limitations. The first study randomized 144 patients 1:1 to either surgical resection or curative RT and found statistically different median survival times of 199 and 300 days, respectively (*p* = 0.04), but the study was heavily limited by the unavailability of CT and PET scans as well as mediastinoscopy and possible under-staging of patients. In fact, 52% of all the surgical patients underwent non-radical (R1 or R2) resection due to unforeseen locally advanced disease. For a long time, these results reinforced existing reservations against surgery for SCLC and have been widely cited as evidence that surgical resection of SCLC is ineffective [148].

The second study randomized 146 patients with central SCLC who achieved an objective response to cyclophosphamide, doxorubicin, and vincristine chemotherapy 1:1 to either surgical resection or conservative treatment. All randomized patients further underwent radiotherapy to the chest and brain. Complete resection was achieved in 83% of the surgical patients, and 19% of patients had pathologic complete remission. The median survival was 15.4 months for the surgical group and 18.6 months for the non-surgical group (*p* = 0.78), leading the study to conclude that the addition of surgery to the multimodality treatment of SCLC had no effect on survival. However, this study was limited by several important factors: an outdated chemotherapy regimen was used, only central tumors were included, RT was administered to both groups, and 35 (7%) of the resected patients had either N1 or N2 disease. These two studies have greatly influenced the surgical management of SCLC since their publications, and their influence remains to this day.

Nevertheless, in 2009, a large registry-based study reported encouraging 5 year survival rates of 49, 33, 6, and 0% in 349 radically resected SCLC patients with stages N0–N3, respectively [149]. One year later, another large, registry-based study reported a 5 year survival rate of 50.3% for 205 radically resected SCLC patients with stage I who underwent lobectomy without RT and 57.1% for 38 patients who underwent both surgery and RT [150]. In 2016, a third study reported 5 year survival rates of 40.4% for 388 radically resected patients with T1–2N0M0 disease who underwent lobectomy without adjuvant chemoradiation and 52.7% for 566 patients who underwent both lobectomy and adjuvant chemoradiation (*p* < 0.01) [151]. All these promising results for early-stage SCLC sparked a renewed interest in surgery for SCLC, and subsequent retrospective studies have found similar promising results. These studies were summarized in a recent meta-analysis that included 13 retrospective studies [152]. The results of this meta-analysis confirmed that surgery significantly improved overall survival when compared to non-surgical treatments (HR: 0.56, *p* < 0.001). Moreover, sub-lobar resections resulted in worse survival than lobectomy (HR: 0.64, *p* < 0.001). Based on this evidence, all guidelines highlight that surgery is justified for selected stage I (T1–2N0M0) SCLC patients and should be carried out as part of a multimodal treatment that includes chemotherapy with or without radiotherapy and a proper multidisciplinary evaluation.

### 5.5. Locally Advanced or Metastatic L-NECs

Systemic therapies and RT for locally advanced or metastatic LCNEC and SCLC follow the guidelines for NSCLC (NSCLC-like LCNEC) or SCLC (SCLC-like LCNEC and SCLC), which are based on high-quality data and strong evidence. Therefore, this part of the management will not be reviewed. Instead, reference is made to applicable guidelines.

Surgery plays a minor role in locally advanced or metastatic LCNEC and SCLC due to the aggressive course of the disease. Possible less-invasive interventions such as bronchoscopic resection and/or stent placement for airway obstruction or fistula should be considered on an individual patient-to-patient basis after a multidisciplinary discussion.

## 6. Follow-Up

Radically resected L-NETs patients should generally be followed up with yearly CT scans for at least 20 years due to the low malignancy of the tumors and tendency of late recurrences. L-NETs patients with locally advanced or metastatic disease and all L-NECs patients should be followed according to applicable guidelines. Treatment factors, including type of surgery, systemic and local treatment, tumor factors such as tumor malignancy and burden, as well as patient factors such as age and comorbidities, should be considered to provide the most appropriate, individualized follow-up care plan.

## Data Availability

No new data were created or analyzed in this study. Data sharing is not applicable to this article.
